# Scheelite type Sr_1−x_Ba_x_WO_4_ (x = 0.1, 0.2, 0.3) for possible application in Solid Oxide Fuel Cell electrolytes

**DOI:** 10.1038/s41598-019-45668-0

**Published:** 2019-06-24

**Authors:** Ahmed Afif, Juliana Zaini, Seikh Mohammad Habibur Rahman, Sten Eriksson, Md Aminul Islam, Abul Kalam Azad

**Affiliations:** 1 Faculty of Integrated Technologies, Universiti Brunei Darussalam, Jalan Tungku Link, Bandar Seri Begawan, BE 1410 Brunei Darussalam; 2 Department of Chemistry and Chemical Engineering, Chalmers University of Technology, Goteborg, SE 41296 Sweden; 3 Faculty of Science, Universiti Brunei Darussalam, Jalan Tungku Link, Bandar Seri Begawan, BE 1410 Brunei Darussalam

**Keywords:** Fuel cells, Electronic properties and materials

## Abstract

Polycrystalline scheelite type Sr_1−x_Ba_x_WO_4_ (x = 0.1, 0.2 & 0.3) materials were synthesized by the solid state sintering method and studied with respect to phase stability and ionic conductivity under condition of technological relevance for SOFC applications. All compounds crystallized in the single phase of tetragonal scheelite structure with the space group of I4_1_/a. Room temperature X-ray diffraction and subsequent Rietveld analysis confirms its symmetry, space group and structural parameters. SEM illustrates the highly dense compounds. Significant mass change was observed to prove the proton uptake at higher temperature by TG-DSC. All compound shows lower conductivity compared to the traditional BCZY perovskite structured materials. SBW with x = 0.3 exhibit the highest ionic conductivity among all compounds under wet argon condition which is 1.9 × 10^−6^ S cm^−1^ at 1000 °C. Since this scheelite type compounds show significant conductivity, the new series of SBW could serve in IT-SOFC as proton conducting electrolyte.

## Introduction

The use of renewable energy and energy conversion and storage have become increasingly important due to the huge demand of energy supply in modern society and emerging ecological concerns in a way which is environmental friendly and low cost^1^. Fuel cells, especially solid oxide fuel cells (SOFC), proton exchange membrane fuel cells, supercapacitors, Li-ion batteries, acousto-optic filter, solid state lasers, photo-catalysts and solar cells etc. are the wide range of technological applications for the energy conversion and storage devices^2–7^. The performance of these devices depends intimately on the properties of their materials.

Because of the high efficiency, fuel flexibility and low pollutant emission, SOFC becomes to be a boundless blessing in alternative energy sector for upcoming generation^8–11^. Oxygen ion conduction requires high activation temperature, which are incompatible with low or intermediate temperature operation. Proton conducting materials can be thermally activated at lower temperatures than oxygen ion conducting ones^12^. At intermediate temperature range (400–700 °C), some perovskite type oxides shows low activation energy and high proton conductivity in H_2_ and H_2_O atmospheres^13–15^. Further development of protonic solid oxide fuel cells operating at intermediate temperature (IT, 400–700 °C) is still important technological challenge^16–19^. The IT-SOFC has proved to be cost effective over conventional high temperature solid oxide fuel cells (HT-SOFC), as IT-SOFC can be manufactured more economically using less expensive stack interconnect materials^20,21^. High-temperature proton conductors have, in general, been found to be oxides with oxygen deficiency in the form of oxygen vacancies, where protons dissolve as hydroxide defects in the oxide at the expense of the vacancies.

Getting the best proton conducting electrolyte material with a highly chemical stability is a great challenge. Synthesis of a highly dense ceramic proton conducting electrolyte materials at low sintering temperature is another major challenge as well. Acceptor doped perovskites are examples of oxides containing both oxygen vacancies and protons. Some of the Ba and Sr containing perovskites exhibit state-of-the-art proton conductivity of about 0.01 Scm^−1^ (e.g. BaCe_0.9_Y_0.1_O_3−δ_)^22–25^. Meanwhile, BaCeO_3_ and BaZrO_3_ based materials exhibit a high conductivity and a good chemical stability^14,26,27^. BaCe_0.7_Zr_0.25−x_Y_x_Zn_0.05_O_3_ proton conducting electrolyte was reported high density and high conductive electrolyte in the intermediate temperature range^15,28^. In the case of the cerates, the basicity of the A cation leads to poor tolerance to CO_2_, rapidly decomposing to form the carbonates at higher temperatures^29,30^. This is a major limitation when one considers using these materials in devices such as fuel cells or hydrogen-separation membranes that operate in hydrocarbon environments.

Recently, alternative proton-conducting materials like acceptor-doped rare-earth materials, MTO_4_, where M = La, Ca, Sr, Ba, Y, Nd, Gd, Tb, Er, Pb, Cd and T = Nb, W, Mo, Mn have been suggested that offer high CO_2_ tolerances based on the scheelite structure^31–36^. Proton conductivity dominates under wet conditions up to temperatures around 1,000 °C with a contribution of p-type electronic conduction, significant under oxidizing conditions above 800 °C. LaNbO_4_-based materials exhibit moderate conductivity while being almost pure proton conductors, and for their stability in CO_2_-containing atmosphere and water vapour environment^31,37^. The highest proton conductivity recorded so far is the material LaNbO_4_ when it contains minor A-site acceptor substitutions, such as Ca_0.01_La_0.99_NbO_4−d_ at 800 °C^38^. It was reported that photoluminescence intensity of mixed tungstate Ca_0.6_Sr_0.4_WO_4_ is higher than for individual tungstate^39^.

Among all scheelite crystals, BaWO_4_ is the most efficient crystal for the development of Raman lasers^40^. The calculations also show BaWO_4_ to be a direct band gap crystal having less dispersive valence and conduction bands and in contrast to other Scheelites crystals. These scheelite-type oxides exhibit a high oxide ion conduction, e.g., Pb_0.9_Sm_0.1_WO_4+δ_ shows a conductivity of ∼2 × 10^−2^ Scm^−1^ at 800 °C, which is comparable to that of YSZ (3.6 × 10^−2^ Scm^−1^ at 800 °C)^36,41^. Ca and Ba doped SrWO_4_ scheelite material were prepared by solid solution and standard wet milling ceramic preparation method and used as optical and photovoltaic applications^42–44^. No work has been reported to use these materials fuel cell devices.

In the current study, Ba doped with SrWO_4_ in A-site of this scheelite composition in order to get sufficient highly density electrolyte material used in SOFC devices. High density coupling with high conductivity will make this material very useful for SOFCs applications. The new mixed ion conducting scheelite Sr_1−x_Ba_x_WO_4_ (SBW) (x = 0, 0.1, 0.2, 0.3), later mentioned as SBW1 (for x = 0.1), SBW2 (for x = 0.2) and SBW3 (for x = 0.3), were prepared by solid state sintering method and examined by using X-ray diffraction (XRD), scanning electron microscopy (SEM), thermogravimetric-differential scanning calorimetry (TG-DSC), Fourier transform infrared (FTIR) and Electrochemical impedance spectroscopy (EIS).

## Result and Discussion

### X-ray powder diffraction

**Figure 1:**
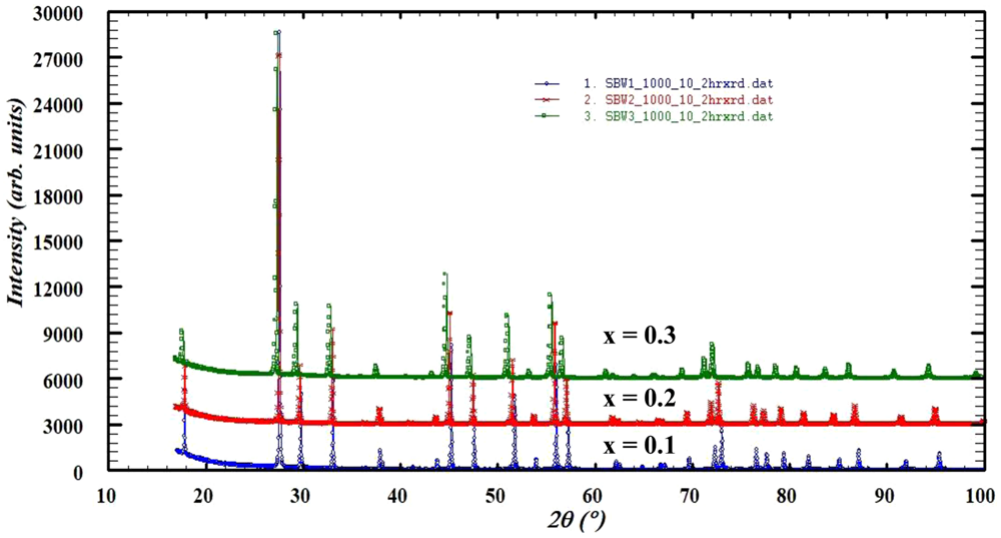
Room temperature XRD patterns of Sr_1−x_Ba_x_WO_4_ (x = 0.1, 0.2 & 0.3).

Figure 1 shows the X-ray diffraction patterns of all SBW compounds (x = 0.1, 0.2 & 0.3) sintered at 1000 °C. In this composition, Strontium (Sr) and Barium (Ba) were A-site components and Tungsten (W) was B-site components, which was fully occupied. Sr^2+^ (ionic radius = 1.44 Å) was replaced by Ba^2+^ (ionic radius = 1.61 Å) as dopant for making A-site 100% occupied. The XRD was carried out as prepared samples. The XRD patterns of all compositions show crystalline nature of the ceramics. No additional or intermediate phases were detected. This suggests that the complete solubility of Ba in the SrWO_4_ crystal lattice at 1000 °C. Earlier studies on these materials the sintering temperature is higher than this study^42,44^.

**Figure 2:**
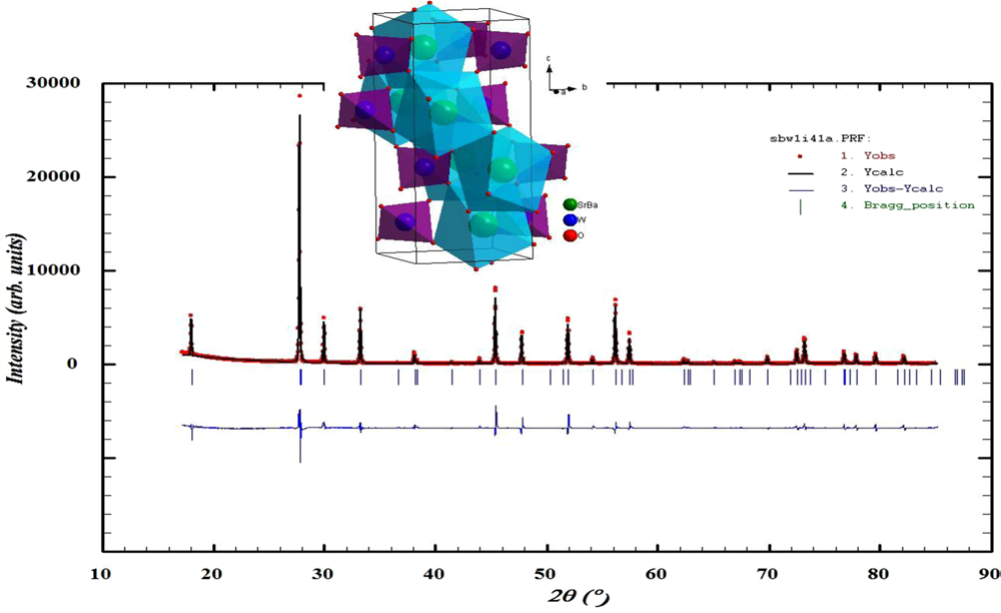
Rietveld refinement profile of Sr_0.9_Ba_0.1_WO_4_, Schematic 3D polyhedral diagram is shown as insert.

The patterns reveal that the Bragg reflections shift to the lower 2θ angle which leads to decrease the unit cell volume because of smaller unit cell volume by doping bigger atomic element (Ba) to smaller atomic element (Sr). The patterns can be indexed as single phase scheelite type tetragonal symmetry in the I4_1_/a space group. Figure 2 shows the refinement of XRD pattern of SBW1 by Rietveld refinement.

Unit cell parameters, densities, volumes and Rietveld refinement factors (R-factors) are listed in Table 1. These scheelites crystallize in the tetragonal symmetry in the I4_1_/a space group where unit cell parameters increase for the doping of Sr by Ba. In 8-coordinated A-site, the ionic radius of Ba^2+^ is 1.42 Å which is higher than Sr^2+^ of 1.26 Å. According to the Vegard’s law, the difference in ionic radii causes the increase in cell volume. Figure 2 shows the Rietveld refinement profile of SBW1; 3D schematic diagram of the tetragonal structure drawn by using Diamond software has been shown in insert. In the scheelite-type ABO_4_ structure with octahedral A cations (Sr/Ba) coordinated with eight oxygen atoms and tetrahedral B cations (W) were connected with four oxygen atoms, which are common binary oxides in both natural and synthetic systems.

**Table 1:** Rietveld refinement analysis of X-ray diffraction data for Sr_1−x_Ba_x_WO_4_ (x = 0.1, 0.2 & 0.3).

Sample Parameters		SBW1 x = 0.1	SLW2 x = 0.2	SLW3 x = 0.3
Space group		I 4_1_/a	I 4_1_/a	I 4_1_/a
Chi^2^ (χ^2^)		10.7	12.3	9.12
Cell parameter (Å)	a = b	5.3803(2)	5.4021(2)	5.4193(2)
	c	11.9059(4)	11.9869(4)	12.0622(5)
Calculated density		6.408	6.396	6.372
Measured density		5.858	6.037	6.109
Relative density (%)		91.41	94.39	95.87
Vol (Å)		344.65(6)	349.81(5)	354.26(2)
No. of fitted parameter		21	21	21
R_f_ - factor		5.62	6.72	7.39
R_p_		14.5	17.2	13.9
R_wp_		18.9	21.8	17.8

### Scanning electron microscopy

**Figure 3:**
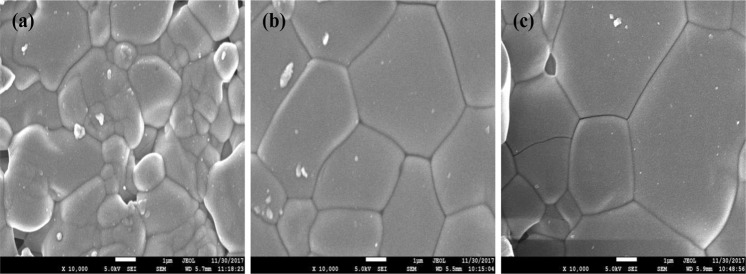
SEM morphology of (**a**) SBW1, (**b**) SBW2 and (**c**) SBW3.

**Figure 4:**
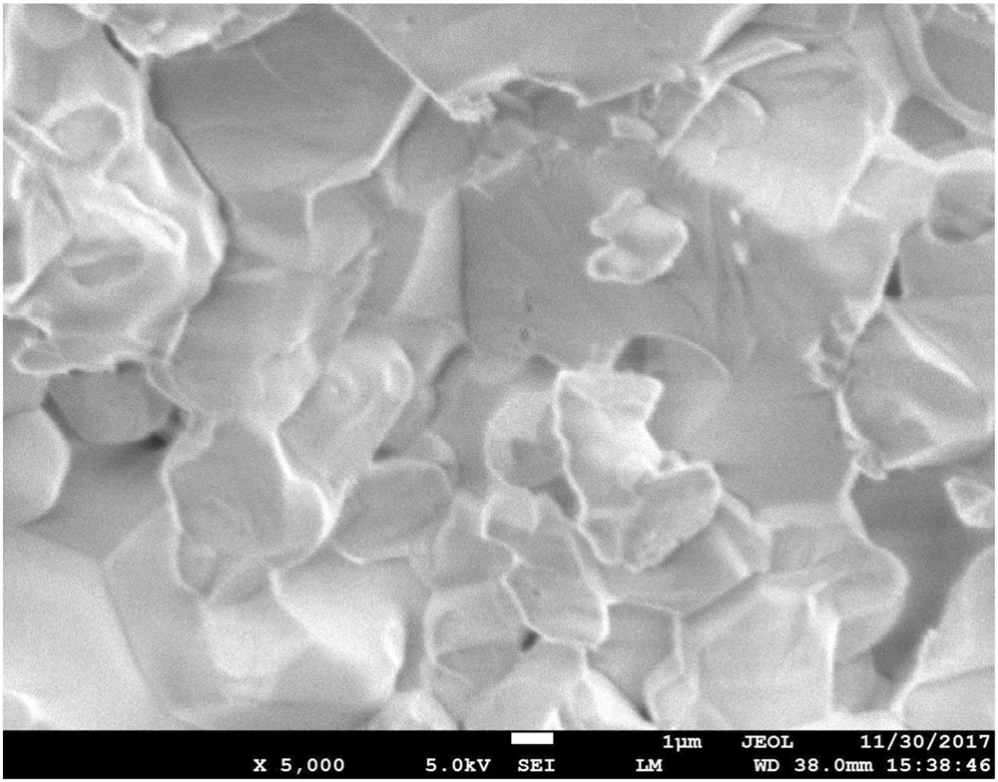
Cross-sectional SEM morphology of SBW1.

**Figure 5:**
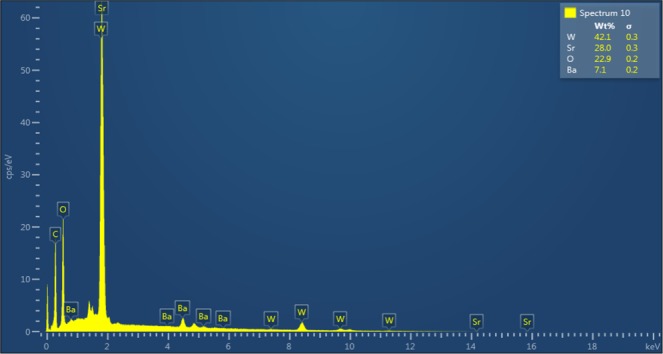
EDX spectra of SBW3.

SEM experiment was carried out to observe the microstructure morphology of the SBW compounds. Figure 3(a–c) shows the surface microstructure of SBW series and Fig. 4 shows the cross-section SEM of SBW1. The surfaces of the samples were smooth and crack free. The grains were connected to each other and well developed making high density materials. The cross-sectional image shows less grain boundaries and less-porous. No trace of secondary or liquid phase was found at the grain boundary region. The grain sizes were also large and compact. These characteristics indicate that non-porous high-density feature of these materials can meet up the required aspect of solid electrolyte. Table 1 show that the relative densities of the samples were around 91 to 95%. The grain sizes of all compositions were around 1–10 µm. In term of protonic conductivity large grain offers less grain boundary resistance which is good for electrolyte. It can be noticed that the increasing Ba concentration in replacement of Sr leads to the decreasing of the density. Figure 5 represents the pattern of compositions by X-ray analysis. Each chemical element has unique electron movement that performs as energy. The figure can be described the intensity of all SBW1 elements. Carbon peaks are also there because of the carbon coating on the samples surface. The element compositions of all three compounds are shown in Table 2 with formula source and EDX source. The results from EDX are reasonably comparable to formula values, because X-ray is effective media to direct the elements of compounds accurately. The uses of X-ray exist in XRD and EDX, this confirm that it is the efficient media to identify the elements of material.

**Table 2:** Compositional distribution of Sr_1−x_Ba_x_WO_4_ (x = 0.1, 0.2 & 0.3) where %F is composition from compound formula and %EDX is composition from EDX.

Samples	Elements	Sr	Ba	W	O
SBW1	% F	30	3.33	33.33	33.33
	% EDX	33.26	3.66	46	17.09
SBW2	% F	26.67	6.67	33.33	33.33
	% EDX	29.80	7.73	44.43	18.03
SBW3	% F	23.33	10	33.33	33.33
	% EDX	26.38	10.6	43.78	19.24

### Thermogravimetric analysis

**Figure 6:**
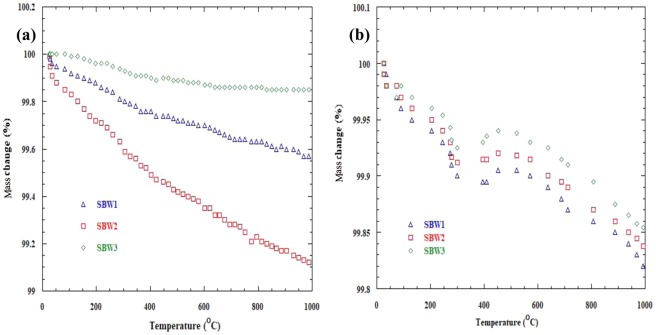
TGA plot of (**a**) dried and (**b**) hydrated Sr_1−x_Ba_x_WO_4_ (x = 0.1, 0.2 & 0.3).

To investigate the hydration behavior through weight loss/gain, thermogravimetric analysis of the samples was performed in the temperature range 25 °C–1000 °C. Figure 6 shows the TGA curves (a) in dried and (b) in hydrated environment for x = 0.1, 0.2 and 0.3, respectively under N_2_. The flow rate was 20 ml/min and the measured sensitivity for all sample compounds were between 0.28–0.58 uV/mW. The weight change was monitored with increasing temperature. During heating, the mass gain started at low temperature (around 30 °C) and got highest at 100 °C which is related to the water uptake from surroundings. In the intermediate temperature range (i.e. 100–600 °C), approximately 60% of theoretically possible protonic defects [OH*] were filled in the intermediate temperature range, which is similar to the result of Ahmed *et al*.^45^.

**Figure 7:**
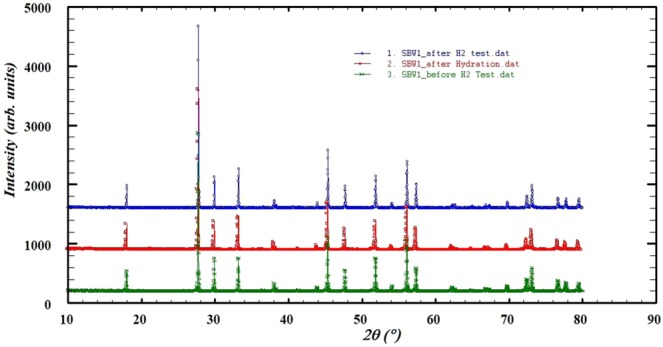
XRD before and after H_2_ and after hydration of SBW1.

All solid oxide proton conductors supposed to hold hydrogen in the intermediate temperature range where H^+^ can conduct following Grotthuss mechanism^46^. The observed maximum mass loss was ≈ 0.43% for x = 0.1, 0.89% for x = 0.2 and 0.16% for x = 0.3 respectively in dried environment. In hydrated environment, initial mass loss occurred from 25 °C to 400 °C. After 400 °C to 500 °C, a mass gain (0.08% for x = 0.1, 0.01% for x = 0.2 and 0.005% for x = 0.3) occurred due to proton absorption, which leads to significant increase the proton content. A mass loss again occurred due to loss of proton at higher temperature. Since hydrogen makes O-H bonds with oxygen, it needs oxygen vacancy. Ba^2+^ doping in replacement of Sr^2+^ at the A-site creates less oxygen vacancies and facilitates the protonic conduction. From neutron diffraction experiments the location of protons can be observed in the structure^47,48^. After hydration and running under H_2_ environment, the XRD was carried out to check the phase stability. Figure 7 shows the XRD curves of SBW1 before and after H_2_ test and hydration process. There was no phase change under H_2_ and hydration^49^.

### FT-IR

**Figure 8:**
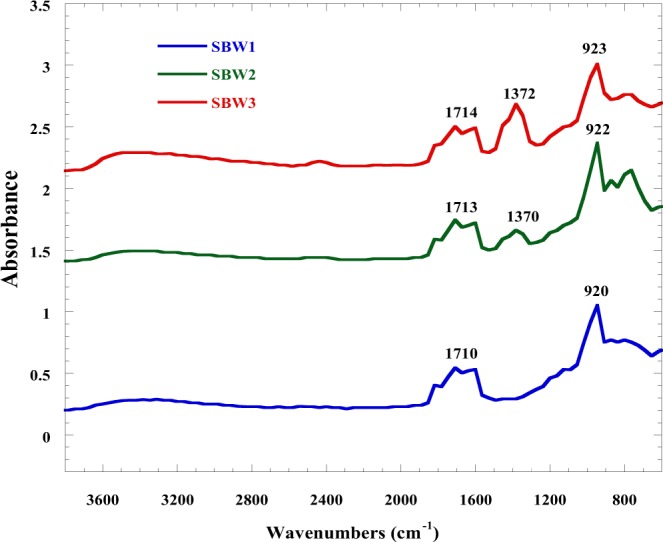
FT-IR spectra of Sr_1−x_Ba_x_WO_4_ (x = 0.1, 0.2 & 0.3).

The FT-IR spectra (Fig. 8) of SBW ceramics measured in the wave number region of 4000 cm^−1^ to 400 cm^−1^ with a resolution of 2 cm^−1^. The characteristic strong and broad absorption bands have two vibration modes around 920 cm^−1^ (SBW1), 922 cm^−1^ (SBW2), and 923 cm^−1^ (SBW3) were assigned to O-W-O anti-symmetry stretching vibrations in [WO_4_] tetrahedron. A sharp but less intense absorption peaks at 1370 cm^−1^ (SBW2) and 1372 cm^−1^ (SBW3) have appeared, which arises due to symmetric bending vibrations in the [WO_4_] tetrahedron. As the concentration of Ba increases in the samples from x = 0.1 to x = 0.3, the absorption band becomes more prominent in SBW3 sample as compare to other samples. Adsorbed water molecules on the surface of the sample at 1710 cm^−1^, 1713 cm^−1^, and 1714 cm^−1^ were also detected for SBW1, SBW2, and SBW3 respectively. The photo catalytic activity and proton conductivity were closely related to the number of -OH groups present on the surface of catalyst because the photo generated holes (h^*^) react with water and generate, OH radicals, which can oxidize the organic pollutants. Therefore, an increase in the number of surface -OH groups could improve the proton conductivity.

### Electrochemical impedance spectroscopy

**Figure 9:**
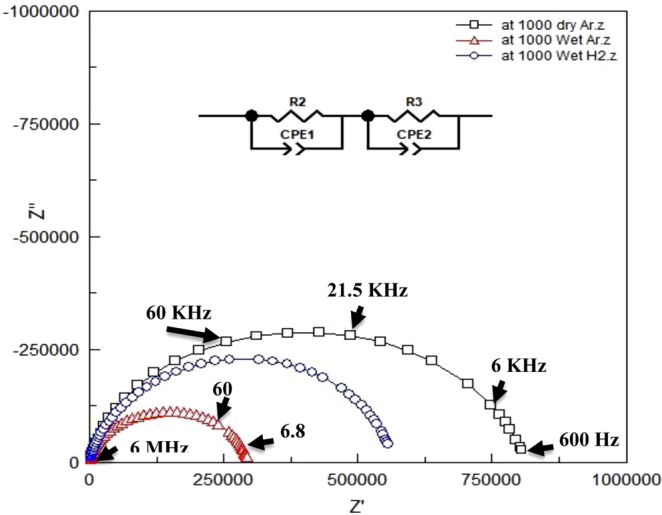
Impedance spectra of SBW3 at 1000 under dry and wet Ar and wet H_2_.

The ionic conduction properties of the SBW series were investigated using AC impedance spectroscopy. Figure 9 shows impedance spectrum of SBW3 recorded at 1000 °C in dry and wet Ar and wet H_2_ condition with bulk and grain boundary (GB) response. A model used to estimate the GB and bulk resistance of the sample compositions. The electrode interface response was excluded from the fitting and the total resistance was the sum of the GB and bulk resistance. In dry Ar, more than one semicircle was observed up to 700 °C in the prepared samples which indicates the existence of GB resistance. The bulk resistance was obtained from the intercept of impedance curve and the real axis (Z^/^-axis) at high frequency. It was difficult to separate bulk resistance from grain boundary resistance above 700 °C although two equivalent circuits was used in series (see Fig. 9 inset). In high temperatures sometimes we need to add inductance at high frequency region to fit the impedance curve with equivalent circuit.

The observed capacitance in the high frequency range was in between 10^−12^–10^−8^ F and in the intermediate frequency range was in between 10^−8^–10^−6^ F which provide the bulk/grain-boundary and sample/electrode response, respectively^50,51^.

**Figure 10:**
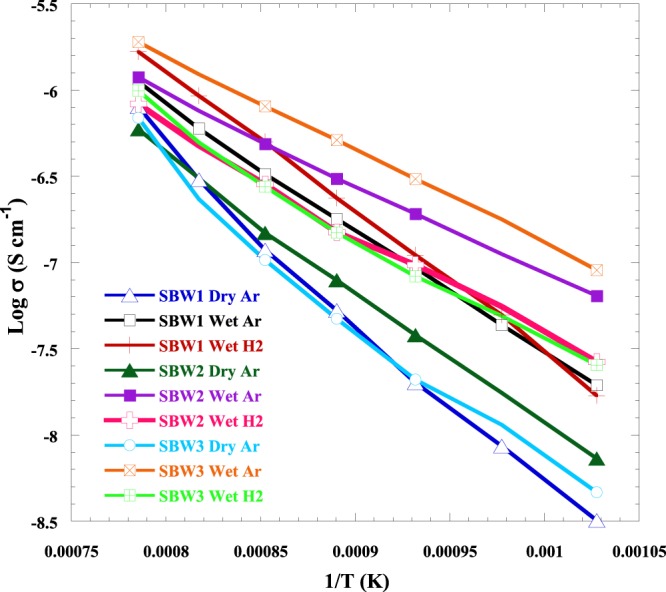
Arrhenius plot of ionic conductivity of Sr_1−x_Ba_x_WO_4_ (x = 0.1, 0.2 & 0.3).

The total conductivities of all 3 samples (x = 0.1, 0.2, 0.3) in wet and dry Ar and wet H_2_ atmosphere is shown in Fig. 10. The total conductivity values were 2.01 × 10^−8^, 9.33 × 10^−8^ and 1.1 × 10^−7^ Scm^−1^ for SBW1, 3.8 × 10^−8^, 1.92 × 10^−7^and 9.69 × 10^−8^ Scm^−1^ for SBW2 and 2.09 × 10^−8^, 3.05 × 10^−7^ and 8.3 × 10^−8^ Scm^−1^ for SBW3, respectively, under dry Ar, wet Ar and wet H_2_ atmosphere. The total (bulk + GB) conductivity increases with the increase of Ba^2+^ dopant i.e. with the decrease of Sr^2+^ content. Conductivity increases in wet Ar in comparison to dry Ar significantly, which indicates the dominant proton conductivity in these samples. the The highest conductivity was 1.9 × 10^−06^ Scm^−1^ for SBW3 at 1000 °C in wet Ar condition. Mixed oxide ion, proton and electron conduction is common in ceramics which can be used as semiconductors, storage materials, fuel cell electrodes and batteries, catalysts and separation membranes. The mixed conduction also depends on the selectivity i.e. the type of conductivity is domination. Moreover, this property depends on the structure and the composition of the materials. In SBW1, protonic conduction was dominating over oxide ion and electron conduction^52^. The mobile ions in these materials are oxide ions and/or protons, sometimes accompanied with electronic conduction^53^. Though the ionic conductivities were lower than typical electrolytes, considering other properties like high density and stability in wet atmosphere we can preliminary select it as a candidate and need to improve its conductivity by modifying its microstructure or doping at A-site of B-site. Table 3 shows the comprehensive comparison of SBW3 with that of the other typical electrolytes. In wet Ar atmosphere, the activation energies (Ea) for the total conductivity were 1.4 eV, 1.03 and 1.07 eV for x = 0.1, 0.2 and 0.3, respectively. Normally all proton conductors show some oxide ion conduction at temperature higher than 700 °C. It could be good to prove/separate conduction type by hydrogen concentration cells. According to the calculation of Irvine *et al*.^51^ and calculation of activation energy, it is possible to understand the nature of the conductivity.

**Table 3:** comprehensive comparison of ionic conductivity of typical electrolytes with this work.

Electrolyte	Ionic Conductivities Scm^−1^	Temperature (°C)	Reference
SBW1	1.1 × 10^−7^	800	This work
SBW2	1.92 × 10^−7^	800	This work
SBW3	1.9 × 10^−06^	1000	This work
BCZY	1.79 × 10^−02^ ~ 2.86 × 10^−02^	300	^56^
YSZ	2.28 × 10^−04^	600	^57^
LSGM	3.6 × 10^−04^	800	^58^
SDC	1.62 × 10^−03^	941	^59^
GDC	1.02 × 10^−03^	941	^59^

Decreasing of Sr^2+^ content in SBW series the activation energy reduces from 1.4 eV to 1.03 eV. The change may be related to the fergusonite – scheelite phase transition, which also occurs within this temperature region. It has been suggested that the change in charge carrier species might be responsible for the difference in activation energy in the scheelite systems. Recent article reported that the activation energy was increased with reduced ionic radius among the LnNb_0.92_W_0.08_O_4.04_ phases; at low temperature, the PrNb_0.92_W_0.08_O_4.04_ showed the lowest Ea (1.34 eV) while LnNb_0.92_W_0.08_O_4.04_ and NdNb_0.92_W_0.08_O_4.04_ had a similar value (1.56 eV)^54^. The variation in activation energy with composition might result from the difference in ionic radius.

## Methods

### Structural measurements

All three compounds, Sr_1−x_Ba_x_WO_4_ (x = 0.1, 0.2 & 0.3), were prepared by solid state reaction method. Stoichiometric amount of BaCO_3_, SrCO_3_ and WO_3_ were mixed in a mortar pestle and grinded using ethanol for proper mixing. The finely ground dried powders were calcined at 700 °C for 10 h with a heating rate of 2 °C min^−1^. Uniaxial hydraulic press was utilized to make 13 mm diameter pellets using 5 ton pressure and subsequently sintered at 900 °C in air for 10 h. The heating and cooling rates were 2 °C min^−1^ and 5 °C min^−1^, respectively. The final sintering temperature is 1000 °C in air for 10 h. The phase characterization was examined by X-ray powder diffraction using Bruker axs-D8 advance diffractometer (CuKα_1,_ λ = 1.5406 Å) in the 2θ range from 10° to 100°. The 60 sec/step counting time and 0.01° step size was used for data collection. The FullProf software was used to refine the obtained data by the Rietveld method^55^. The morphological characteristic of the prepared electrolytes were examined using FEG-SEM (JSM-7610F). Sample chamber evacuated before colleting SEM images.

### TGA and FT-IT measurements

The weight change with increasing temperature was conducted by thermogravimetric analysis. The samples were introduced to hydration furnace in in wet N_2_ atmosphere. N_2_ gas was passed through water to make it wet which gives about 3–5% H_2_O to flow with N_2_ gas. The hydration steps were at 800, 600, 400, 200 and 150 °C with residence time of 2, 2, 48, 2 and 55 h, respectively. The samples were heated at 200°C/h until 800 °C and cooled at 10 °C/h to 150 °C. The hydrated samples were investigated by a NETZSCH thermogravimetric analyzer. Nitrogen flowed constantly in the TGA at 20 mL/min. Fourier transform infrared (FTIR) spectra were recorded by PerkinElmer Spectrometer for diffuse infrared spectroscopy in air at room temperature. Powder samples were subtracted with a run with optically transparent KBr, as a reference.

### Electrochemical measurements

The electrochemical properties were examined using a Solartron electrochemical impedance analyzer with frequency response analyzer connected to the fuel cell using ProboStat (NorECs, Norway). The impedance data was collected in the frequency range from 1 mHz to 6 MHz using applied sine wave amplitude of 1 V rms. Platinum electrodes were used on both sides of the sample (13 mm diameter and 0.5 cm^2^ electrode area) for the impedance measurements. Impedance data were collected from 1000 to 150 °C with the steps of 50 °C under dry and wet Ar atmosphere in cooling cycle. Ar gas passed through 2 beds of P_2_O_5_ desiccant to find dry Ar whereas Ar gas flowed through water at 22 °C (p(H_2_O) = 0.026 atm) to find wet Ar atmosphere. After reaching each temperature, 30 minutes isothermal time was given to ensure stability before recording impedance spectra. Z-View (Scribner Associates Inc.) refinement program was used to fit and analyze the experimental impedance data. The brick-layer model of grain boundaries was assigned to represent the electrical response of the sample compositions. Each curve from the experimental data interpreted a parallel sequence of a resistance (R) and a constant-phase element (CPE). The resistance could not be extracted reliably due to high impedance at lower temperature (200 °C),.

## Conclusion

In this research study, the main focus was to develop Scheelite type Sr_1−x_Ba_x_WO_4_ (x = 0.1, 0.2 & 0.3) electrolytes for SOFC applications. SBW electrolytes were successfully synthesized by solid state sintering method and characterized by using XRD, SEM, TGA and EIS. Fullprof software was used to analyze XRD data in the Rietveld method which showed a tetragonal scheelite structure in the I4_1_/a space group. The unit cell parameter increases with Ba-doping. SEM morphological images showed a high density and non-porous materials which is important for electrolyte application. Ba doping increases the grain size. TGA analysis showed a significant proton uptake at the intermediate temperature range. In terms of conductivity, all compound shows low ionic conductivity. SBW3 exhibited the ionic conductivity of 1.9 × 10^−6^ S cm^−^¹ at 1000 °C under wet argon condition. From the obtained results it indicates that these kinds of materials have a very good microstructure and significant conductivity with good stability which can be applied as electrolyte materials for IT-SOFCs.
